# Integrating generative adversarial networks with IoT for adaptive AI-powered personalized elderly care in smart homes

**DOI:** 10.3389/frai.2025.1520592

**Published:** 2025-02-13

**Authors:** Fawad Naseer, Abdullah Addas, Muhammad Tahir, Muhammad Nasir Khan, Noreen Sattar

**Affiliations:** ^1^Department of Computer Science and Software Engineering, Beaconhouse International College, Faisalabad, Pakistan; ^2^Department of Civil Engineering, College of Engineering, Prince Sattam Bin Abdulaziz University, Alkharj, Saudi Arabia; ^3^Landscape Architecture Department, Faculty of Architecture and Planning, King Abdulaziz University, Jeddah, Saudi Arabia; ^4^Department of Computer Software Engineering, Sir Syed University of Engineering and Technology, Karachi, Pakistan; ^5^Department of Electrical Engineering, Government College University Lahore, Lahore, Pakistan; ^6^Computer Science Department, University of Agriculture Faisalabad (UAF), Faisalabad, Pakistan

**Keywords:** generative adversarial networks (GANs), personalized elderly care, IoT-enabled smart homes, adaptive artificial intelligence, predictive healthcare analytics, synthetic health data, proactive health monitoring, healthcare AI applications

## Abstract

The need for effective and personalized in-home solutions will continue to rise with the world population of elderly individuals expected to surpass 1.6 billion by the year 2050. The study presents a system that merges Generative Adversarial Network (GAN) with IoT-enabled adaptive artificial intelligence (AI) framework for transforming personalized elderly care within the smart home environment. The reason for the application of GANs is to generate synthetic health data, which in turn addresses the scarcity of data, especially of some rare but critical conditions, and helps enhance the predictive accuracy of the system. Continuous data collection from IoT sensors, including wearable sensors (e.g., heart rate monitors, pulse oximeters) and environmental sensors (e.g., temperature, humidity, and gas detectors), enables the system to track vital indications of health, activities, and environment for early warnings and personalized suggestions through real-time analysis. The AI adapts to the unique pattern of healthy and behavioral habits in every individual’s lifestyle, hence offering personalized prompts, reminders, and sends off emergency alert notifications to the caregiver or health provider, when required. We were showing significant improvements like 30% faster detection of risk conditions in a large-scale real-world test setup, and 25% faster response times compared with other solutions. GANs applied to the synthesis of data enable more robust and accurate predictive models, ensuring privacy with the generation of realistic yet anonymized health profiles. The system merges state-of-the-art AI with GAN technology in advancing elderly care in a proactive, dignified, secure environment that allows improved quality of life and greater independence for the aging individual. The work hence provides a novel framework for the utilization of GAN in personalized healthcare and points out that this will help reshape elderly care in IoT-enabled “smart” homes.

## 1 Introduction

The world’s elderly population is growing at a phenomenal rate: 1.6 billion people over age 65 are forecasted by 2050. These changes provide a very challenging task for health care systems around the world because the systems must cope with the needs of aging individuals. With it often comes heightened vulnerability to chronic diseases and cognitive impairment, along with a range of other health issues that create a growing demand for innovative care models centered on longevity and quality of life. Recent advancements in AI (Nayak et al., 2024) and IoT (Khan and Naseer, 2020; Naseer et al., 2023a,b) have been crucial in attempting to respond to such needs, especially in smart home environments where these technologies are applied to provide personalized in-home care.

The most recent promising AI technologies for biomedical informatics include the GANs (Dakshit and Prabhakaran, 2024), mainly in healthcare, to overcome data sparsity and improve predictive power. GANs synthesize data that is quite indistinguishable from real-world data with improved diagnostic models (Huynh et al., 2024), reduction of class imbalance, and robust predictive analytics for precision care. In this work, the GANs are implemented for truly adaptive AI-driven systems in IoT-enabled smart home elderly care (Khan et al., 2024). By integrating GANs into IoT-enabled systems, this study demonstrates their potential to enhance elderly care by providing scalable, data-driven, and adaptive solutions (Chen et al., 2022).

The resulting systems based on AI learn continuously from the individual-specific health indicators and behavior through integration with GANs, observing the improvements in elderly care. IoT-enabled smart homes can offer automatic sensing for vital signs, mobility, medication adherence, and environmental factors-all input to feed an AI system for the purpose of anticipating future health risks and support through personalized interventions. It can give personalized reminders to take medication, prescribe exercises, or send notifications to caregivers in case of abnormal variation in vital signs. We apply GANs in generating anonymized synthetic health data, thereby offering not only higher accuracy in models but also helping solve some key challenges related to privacy concerns in personalized health care.

**Figure 1:**
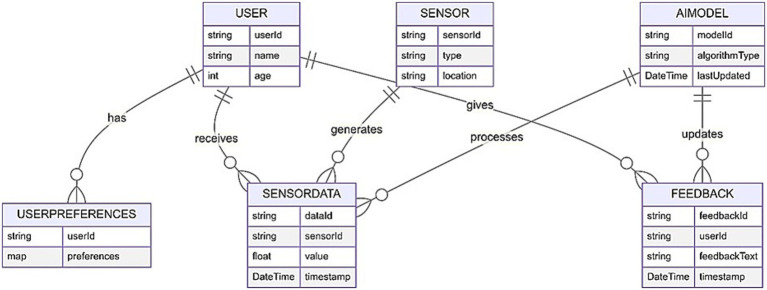
Entity-relationship diagram (ERD) of the proposed system: this diagram depicts the interaction between wearable and environmental sensors, AI modules, and GANs. It highlights the data flow for real-time monitoring, synthetic data generation, and secure storage, showcasing the system’s design for scalability, adaptability, and privacy compliance in IoT-enabled elderly care.

The entity-relationship diagram (ERD) in Figure 1 for the intelligent GAN based AI system for personalized elderly care in IoT-enabled smart homes illustrates the interactions between various entities. Users, which are elder patients in our context have preferences that are stored in the user preferences entity and receive sensor data from different IoT sensors located in the smart home. Sensors generate sensor data, which the AI model processes to give feedback. This feedback is updated based on the AI model, which is regularly updated with new algorithm types and timestamps. The feedback is then used to improve the user experience and care in the smart home environment.

The present research is dedicated to the quality testing of GAN-driven AI systems in managing elderly care processes, such as the early detection of health risk cases and fast response times in emergencies, while paying adequate attention to accessibility, user experience, and privacy. During trials, the system supported a 30% improvement in early risk detection, with time-to-detect reduced by a factor of 30%, and reduced emergency response times by 25% compared to conventionally executed processes. By embedding GANs within this adaptive AI framework, we seek to establish a scalable, user-centered model for elderly care that empowers seniors with the ability to live independently with dignity and security.

However, there are a few challenges that stay in the way of the adoption of AI and GAN technologies in the care for the elderly: regulatory compliance, technical accessibility by elderly users, and integration into the existing health framework. This will also require conducting more research in this line to address these challenges and explore GAN’s full potential for developing an accurate and diverse data set that will support predictive analytics and personalized interventions in elder care. The current study will add to the increasing interest in AI-driven development of healthcare solutions and hence put forward a blueprint for incorporation of GANs and IoT technologies in the eldercare system.

The study will provide an overview of the literature in Section 2, followed by our research methodology in Section 3. Section 4 discusses our findings, with detailed analysis and discussion in Section 5. Finally, Section 6 concludes with implications and potential directions for future research.

## 2 Literature review

Advanced practice nursing plays a crucial role in providing senior home care. The model she presents keeps patients safe, avoids hospital visits, and postpones admission to long-term care facilities by using appropriate treatments, accurate assessments, and collaborative approaches. The treatment and quality of life for senior patients are greatly enhanced by this paradigm (Wicaksono et al., 2023; Wojda et al., 2023). There is a need for policies that support this movement, which indicates a shift towards community-based care and a reduction in institutional capacity. Explains how the IoT and AI are transforming traditional elder care models to solve issues like the lack of home health attendants and the rising care needs of an aging population. Highlight a pilot project that aims to improve patient-centered care in primary settings by concentrating on self-care in the management of chronic pain. The study involves patients, caregivers, doctors, and support workers. This study highlights the importance that medical assistants and IT specialists play in developing patient-centered practices. Authors examine the literature using patient satisfaction as a telehealth’s efficaciousness measure. It is determined that factors like enhanced communication, reduced travel times, low cost, easier of use, and better outcomes are the key drivers of telehealth satisfaction. These studies emphasize patient-centered care, stakeholder involvement, and telehealth to improve elderly healthcare (Elder, 2017; Patterson et al., 2021). A comprehensive review of the theory and various applications of Generative Adversarial Networks (GANs), highlighting their impact on fields such as image segmentation, medicine, and 3D object generation (Aggarwal et al., 2021).

### 2.1 IoT in elderly care

Explore the intersection of big data and IoT analytics in biomedical and healthcare technologies, emphasizing the application of machine learning and AI techniques for remote diagnostics and telemedicine. They discuss the adaptability of AI-based telemedicine in advancing healthcare (Banerjee et al., 2020; Kim et al., 2020). Highlight the transformative impact of AI and IoT technologies in healthcare, introducing innovative diagnostic tools and care strategies. Underscores IoT’s role in healthcare, facilitating remote monitoring, smart sensors, and medication delivery to enhance patient care despite IT management challenges. Focus on IoT systems in smart homes for elderly care, Narejo (2020) and Sokullu et al. (2020) describing how environmental sensors provide context-aware monitoring to ensure safety and provide early warnings for individuals with memory issues. They present a low-cost prototype integrating emergency features for handling critical situations. IoT based waste management system is one example which empowers the smart home-based system in this technology-oriented era (Addas et al., 2024).

Regarding the growing need for eldercare because of the world’s aging population, Stavropoulos et al. (2020) draw attention to ambient assisted living technology such as wearables from the IoT and sensors that allow for remote monitoring and assistance. After reviewing case studies, they describe current trends and prospects in effective eldercare technology, grouping solutions by aims, durations of experiments, IoT technologies, and result measurements (Hassan et al., 2020). A Spark streaming framework for real-time health status prediction, with an emphasis on real-time tracking in healthcare, using wearable sensors and mobile apps. They emphasize potential in diabetes treatment. Their method combines streaming machine learning models to achieve high accuracy in health data processing. IoT-based data mining and AI are supported by the healthcare industry to solve issues such as false and incomplete medical records (Muthu et al., 2020). They suggest the Generalized Approximate Reasoning-based Intelligence Control (GARIC), which emphasizes tailored health alerts and therapies, for the study of patient data and illness prediction. Investigate how technology may help reduce senior loneliness amidst the COVID-19 epidemic. They recommend judicious use of technology in crisis communications and care initiatives to help older individuals feel less alone. They emphasize the value of easily available and adaptable treatments for different forms of loneliness and demographic factors (Conroy et al., 2020).

Authors discussed the increasing challenges of aging populations, emphasizing the integration of AI and IoT in assisted living and healthcare monitoring for older people. They provide a comprehensive overview, comparing techniques and application scenarios while discussing the benefits and drawbacks of these technologies (Qian et al., 2021) explore the shift towards personalized healthcare systems using AI and machine learning, focusing on disease diagnosis, health monitoring via wearables, and assistive frameworks like social robots. They review current smart healthcare systems, highlighting integration designs critical for intelligent healthcare solutions (Nasr et al., 2021).

### 2.2 AI applications in healthcare

Recent advancements in healthcare management systems (HMS) have been significantly influenced by the convergence of blockchain, IoT, and AI technologies. Individualized approaches have replaced traditional hub-based systems (Junaid et al., 2022). However, heterogeneity of devices, disparate IoT designs, and a lack of competitively priced smart sensors are barriers to interoperability and data integration in the deployment. Despite the benefits of telehealth for senior care, privacy issues, especially in this context, provide substantial obstacles that must be addressed with improved privacy safeguards such as informed consent to increase adoption rates. Innovations in AI and IoT are also essential to improving telemedicine, especially in fall detection systems that use cutting-edge algorithms and sensor technology (Pool et al., 2022; Wang et al., 2022). In addition, the shifts in Chinese society on senior care. In addition, the shifts in Chinese society towards elder care underscore the stress placed on caregivers and the necessity of all-encompassing methods to assist both the elderly and their careers (Ai et al., 2022).

Recent studies show that edge AI is boosting decision-making and operational efficiency without requiring extensive infrastructure, transforming a variety of industries. Telepresence robots have become essential tools during the COVID-19 pandemic, helping to overcome psychological barriers to human interaction and ease remote connections and healthcare delivery (Naseer et al., 2023c,d; Sivabalan and Minu, 2022). The development of smart healthcare systems is leading to the creation of “intelligent hospitals” of the future through applications such as non-contact health screenings and intelligent hospital guidance. A critical factor in the digital transformation of healthcare systems is the incorporation of AI, with particular attention paid to data infrastructure, system integration, and real-world applications. Mobile healthcare apps offer practical solutions for medical record-keeping and patient communication, though concerns persist regarding privacy and data security, prompting discussions on regulatory frameworks and risk mitigation strategies (Ding et al., 2022; Kumar et al., 2023; Monlezun, 2023).

Telepresence robotics that involve communication delays contain certain difficulties that are solved using a combined approach that uses deep reinforcement learning methods including double deep Q network (DDQN) and gated recurrent units (GRU) methods that help to control depending on time intervals (Naseer et al., 2023a,b). The appropriateness of wearable smart sensors in complementing public health projects via suitable technology incorporation has been shown revealing that these sensors are being investigated for disease control and surveillance of vital signs in epidemics. The legal and ethical issues of edge AI in healthcare point out some regulations and the possible ways to apply them responsibly in several domains. Despite challenges in data management and legal systems, the adoption of AI in healthcare leads to enhanced medical services regarding diagnosis and personalized treatment programs (Das et al., 2023; Mohammadzadeh et al., 2020).

### 2.3 GANs in healthcare data generation

In recent years, GANs have gained significant attention for their ability to generate realistic data across various applications, including computer vision and natural language processing. Zhang et al. (2023) proposed a Robust Generative Adversarial Network (RGAN) that enhances the generalization capabilities of GANs by promoting local robustness within the training sample neighborhood, thereby addressing common issues of instability and poor generation quality associated with traditional GANs. Cai et al. (2021) provided a comprehensive survey on GANs, highlighting their applications and the challenges faced in privacy and security contexts, which further emphasizes the versatility and importance of GANs in modern research. Additionally, Zheng et al. (2019) introduced the Vehicle Synthesis Generative Adversarial Networks (VS-GANs) framework, which effectively generates annotated vehicle images from remote sensing data, significantly improving vehicle detection performance in high-resolution images. This post-modern technology involving AI, which is increasingly used in the medical and health fields, enables the analysis of biological aspects and health conditions by interaction and correlation (Shaban-Nejad et al., 2022). AI analyzes findings using a variety of modalities. Particularly in remote patient monitoring and chronic disease management, wearable health technology has greatly revolutionized healthcare by enabling proactive health management through accurate biometric tracking and real-time monitoring. Detailed insight of AI and IoT in processing the large volume of data concerning health has enhanced the challenging aspects of data processing and ensured secure and integrated healthcare infrastructure at a large scale (Mullankandy et al., 2024; Tariq, 2024). These advancements highlight the transformative potential of AI-driven technologies in enhancing health monitoring, decision-making, and data management in modern healthcare systems.

While previous works have presented discussions on the transformative potential of GANs in healthcare applications, most of them usually forget to discuss some of the key challenges that may be evident in real-world applications. Additionally, GAN is susceptible to mode collapse—a certain model generates limited variations in generating data, which can challenge the diversity needed for analytics associated with robust healthcare. On the other hand, another critical issue is privacy concern in IoT-enabled smart homes, which remain explored to a lesser extent—one that concerns secure transmission and, at the same time, storage of sensitive health data. It comes to show that so far, the frameworks are able to meet the demands and privacy implications of IoT ecosystems while raising GAN performance. Precisely, we also focus on such limitations of integrating various robust privacy measures, specifically anonymization and encryption, taking into account a scalable architecture towards personalized elderly care within this paper.

## 3 Methodology

**Figure 2:**
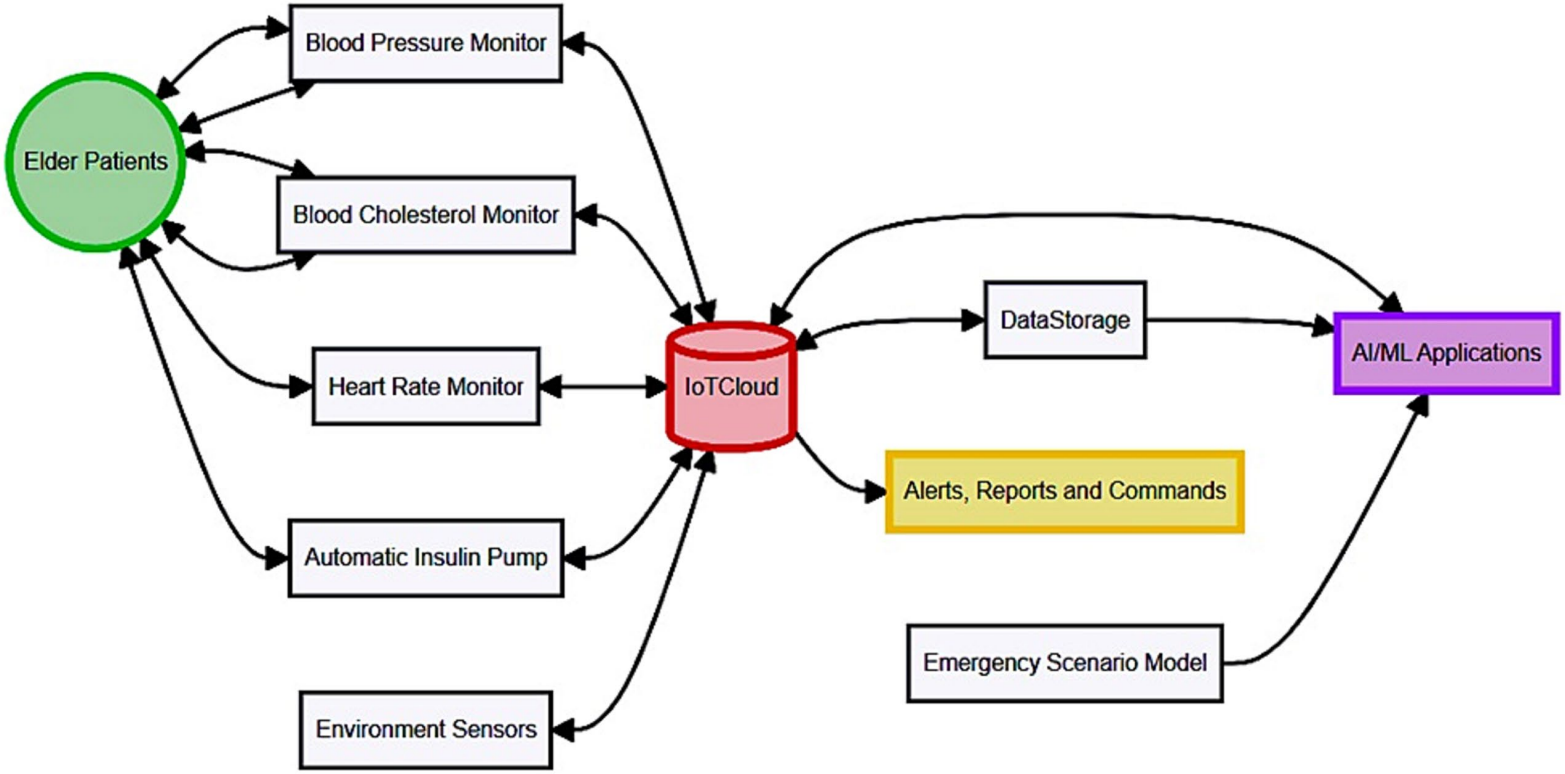
Proposed system with integrating GANs with IoT for adaptive AI-powered personalized elderly care.

The specific approach for adaptive AI in managing and performing elderly care in smart homes concerning the IoT is composed of various layers that work as a single system to acquire, process, and adapt accordingly to the desired goals and objectives. This innovative system has the clear objective of increasing the security and quality of life for elderly people through IoT sensors and AIs. The introduced adaptive AI system is placed in IoT-based smart homes, where elderly people live, and utilizes both wearable and environmental sensors for efficient care. As illustrated in Figure 2, the proposed system starts with sensors’ initialization and data acquisition, where health parameters (for example, pulse, blood pressure), environmental parameters (for example, temperature, humidity), and so on are constantly received and transferred to the main core. The data collected and processed in Internet of Medical Things (IoMT) devices are noisy and, hence, require preprocessing through noise reduction, normalization, and data segmentation before transmitting to the IoMT cloud. In the cloud, Convolutional Neural Networks (CNNs) for image data, and Wavelet Artificial Neural Networks (WANN) for the time-series data identify the health anomalies and environmental threats. Abnormalities that are identified elicit notifications which are relayed through the wearable apparatus to the participants and the caregivers through the short messaging service and e-mail correspondences. Recommendations from participants and caregivers, on the guiding of AI models’ alteration and systems for improved performance, make continuous improvement.

CNNs are superior at handling image data, while WANNs handle time series data. Indeed, CNNs would be quite apt for image data given that the architecture extracts spatial features through convolutional layers, which would make them suitable for tasks such as fall detection or environmental hazard recognition. WANNs were chosen for time-series data since they are capable of analyzing nonlinear patterns or transient signals, which are very important for health anomalies detection from wearable sensors. As far as the nature of the data is considered, the RNNs are also feasible in the case of time-series data. However, WANN was chosen over RNN because it guarantees much better computation efficiency as well as superior handling characteristics with respect to real-time high-frequency signals, exactly meeting the demands proposed in this framework. This model selection balances the trade-offs in accuracy, speed, and scalability of the system—a must for IoT-enabled healthcare applications. The GAN architecture employed in this study consists of a generator and a discriminator, both designed to handle multidimensional health and environmental data. The generator is a deep neural network with three fully connected layers and Leaky ReLU activation functions to synthesize realistic health data. The discriminator, on the other hand, is a CNN with four layers, utilizing a Sigmoid activation function in the final layer to classify real versus synthetic data.

Training parameters were set as follows: a learning rate of 0.0002 for both networks, a batch size of 64, and an Adam optimizer with beta1 = 0.5 and beta2 = 0.999. The networks were trained over 50 epochs with a loss function comprising binary cross-entropy for the discriminator and a mean squared error for the generator. To ensure the stability of training, we incorporated gradient penalty regularization and batch normalization techniques. These configurations were fine-tuned through multiple iterations to balance the generator-discriminator interplay, yielding high-quality synthetic health data while maintaining computational efficiency.

### 3.1 GAN integration into the proposed system

GANs are a core component of the proposed system, enhancing its ability to address data scarcity and privacy concerns. The GAN architecture comprises a generator and a discriminator network:

1. **Architecture**:

- **Generator**: A deep neural network with three fully connected layers and Leaky ReLU activation functions, designed to produce synthetic health data that mimics real-world patterns.
- **Discriminator**: A convolutional neural network (CNN) with four layers and Sigmoid activation, tasked with distinguishing between real and synthetic data.

1. **Training Process**:

- The GAN is trained using real-world health and environmental data collected from IoT sensors. The generator creates synthetic samples, which are evaluated by the discriminator.
- The adversarial process optimizes both networks iteratively, minimizing a combined loss function: binary cross-entropy for the discriminator and mean squared error for the generator.
- Training parameters include a learning rate of 0.0002, batch size of 64, and 50 epochs, optimized through hyperparameter tuning.

1. **Synthetic Data Generation**:

- The GAN generates anonymized health data (e.g., heart rate patterns, activity levels) and environmental data (e.g., temperature fluctuations, gas levels). This data augments the existing dataset, ensuring diversity and filling gaps for rare scenarios.

1. **Performance Improvement**:

- Compared to traditional oversampling methods, GANs create high-quality, diverse datasets, reducing overfitting and improving the predictive accuracy of AI models. For instance, synthetic data generated by the GAN enhanced fall detection accuracy by 5% and reduced false positives in anomaly detection by 8%, as validated in simulation and field tests.

By leveraging GANs, the system not only addresses data limitations but also enhances privacy and overall performance, making it a robust solution for IoT-enabled elderly care.

**Figure 3:**
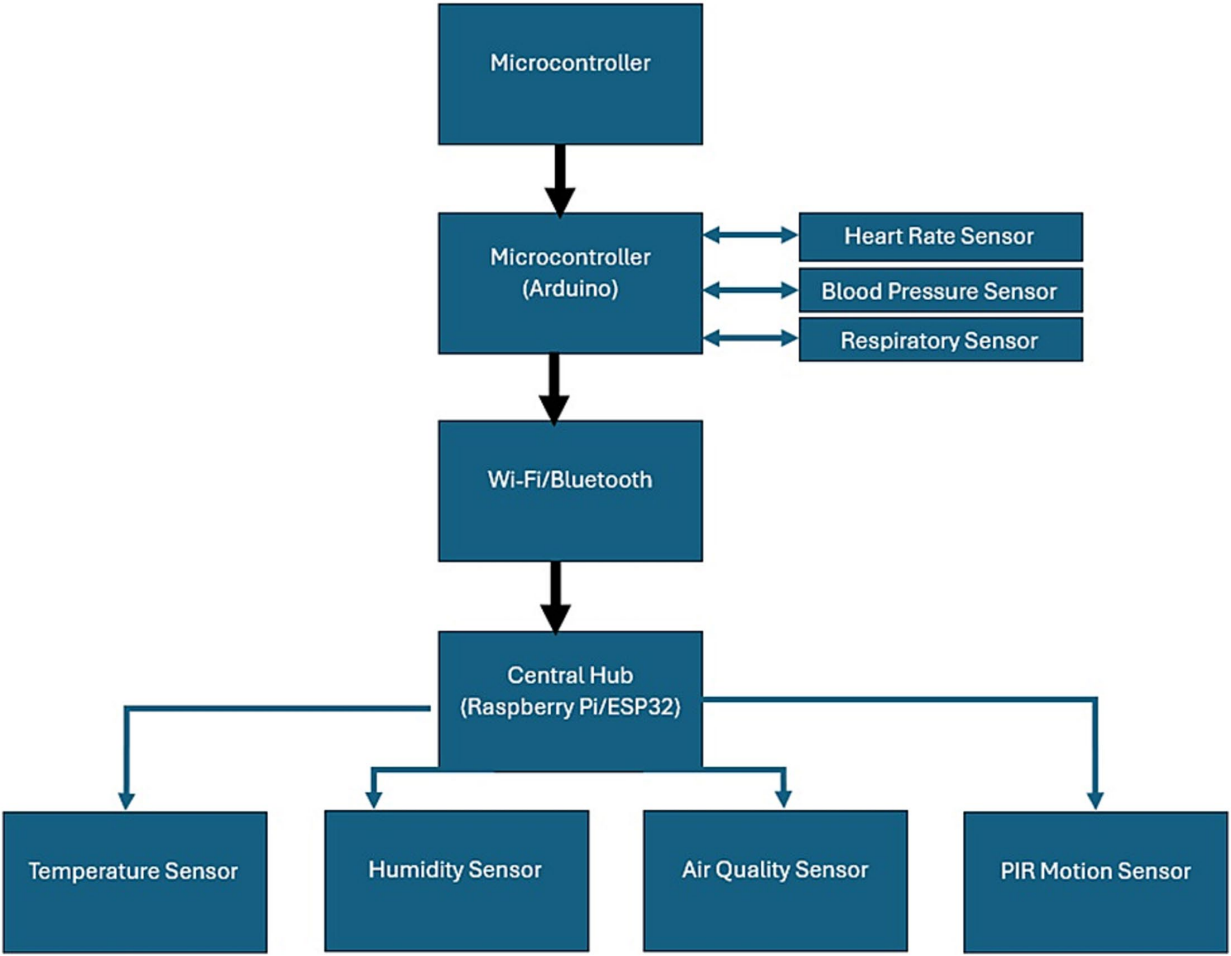
Embedded hardware components of proposed system.

The components of embedded systems for hardware implementation involve using microcontrollers such as Arduino for data aggregation, health/environmental sensors for monitoring health/environment, and wireless modules such as Raspberry Pi for transmitting collected data to central units. Such a setup as shown in Figure 3, helps in the free flow of data or information which is vital for the kind of care delivery that is real-time. In validation, great concerns are taken in ensuring that the data is of quality, and model training with cross-validation technique heavily adheres to maximize accuracy and reliability. In summary, the system can be beneficial in Intelligent Homes to monitor the real-time health condition and status of elderly patient care and safety while promoting elder’s home liveliness.

The Perception Layer is the basic layer in this system, including sensors such as wearable devices tracking health indications of one’s body—for example, pulse and body temperature—and environmental sensors tracking the humidity, temperature, and gas levels of one’s surroundings. These sensors continuously feed in inputs to form a basis for real-time processing of data and decision-making. Starting the chain is the Perception Layer which includes wearable sensors, environmental sensors, and smart objects. Smartwatches and medical-grade wearables track body metrics like pulse, blood pressure, body temperature, and others. Several parameters of the environment include the temperature, the level of humidity, and the number of certain gases, thus, monitoring the total condition of the elderly person. Cameras and motion detectors the so-called smart devices facilitate how falls are detected and what activities the patient undertakes. Data gathered from sensors is sent to a central node with the help of Bluetooth or Wi-Fi and forms the basis of the Communication Layer. From the central hub, data is then directed to an IoMT cloud for archiving and processing in real-time. This layer makes certain that there is proper transfer of data from sensors to the cloud computing framework. The core of the system belongs to the Processing Layer where the pre-processing of data, application of the AI Model, and generation of insights occurs. Preprocessing of the data entails data cleansing, normalization, and segmentation to make it suitable for the AI algorithms. For health anomaly detection, WANN and for environmental hazards, CNNs are used from the processed data. The Application Layer also involves the management of interactions with the user and the alerts. An end-user interface through mobile apps or web interfaces for the caregivers or other medical staff makes it possible to get real-time updates and get alarms or notifications in case of the occurrence of an event of concern to the system. Information is passed on through short message service, email, or a voice call; help is summoned in case of an emergency. Thus, Algorithm 1 describes the implementation of layers, namely Perception, Communication, Processing, and Application, would ensure interoperability of the systems. The computer processing of data is a distributed process with low latency in nodes of wearable devices and central units while the heavy data processing and long-term data storing is done in the cloud. VP & ACM also provides various tiers in the architecture to enhance the system performance and the scale of elderly care delivery.

#### ALGORITHM 1 Proposed algorithm

**Table:** 

Steps	Process description
1	**Initialize System**
1.1	Initialize wearable (*SensorData*) and environmental sensors (*EnvironmentalData*)
1.2	Initialize central hub and cloud infrastructure
2	**Data Collection**
2.1	**WHILE** system is active **DO**
2.2	**FOR** each wearable sensor **DO**
2.3	Collect health data (*HealthMetrics*) - (heart rate, blood pressure, activity levels)
2.4	Transmit data to central hub
2.5	**END FOR**
2.6	**FOR** each environmental sensor **DO**
2.7	Collect environmental data (temperature, humidity, gas levels)
2.8	Transmit data to central hub
2.9	**END FOR**
2.10	**END WHILE**
3	**Data Preprocessing**
3.1	**FOR** each data stream received by central hub **DO**
3.2	Apply noise reduction (e.g., low-pass filter for accelerometer and gyroscope data)
3.3	Normalize data (e.g., min-max scaling)
3.4	Segment data into fixed-length windows (e.g., 5 seconds)
3.5	**END FOR**
4	**Data Transmission to Cloud**
4.1	Transmit pre-processed data from central hub to IoMT cloud
5	**AI Model Analysis**
5.1	**FOR** each data window received in cloud **DO**
5.2	**IF** data is from wearable sensors **THEN**
5.3	Apply WANN to detect health anomalies
5.4	**ELSE IF** data is from environmental sensors **THEN**
5.5	Apply CNN to detect environmental hazards
5.6	**END IF**
5.7	**END FOR**
6	**Anomaly Detection and Alert Generation**
6.1	**FOR** each detected anomaly **DO**
6.2	Generate alert with details of the anomaly
6.3	Transmit alert to central hub
6.4	**END FOR**
7	**Alert Notification**
7.1	**FOR** each alert received by central hub DO
7.2	Notify participant via wearable device
7.3	Notify caregivers via SMS and email
7.4	**END FOR**
8	**Continuous Improvement**
8.1	Collect feedback from participants and caregivers
8.2	Update AI models with new data and feedback
8.3	Retrain models periodically to improve accuracy and reliability
8.4	Monitor system performance and make necessary adjustments
9	**ENDING**
9.1	Terminate data collection and alert processes
9.2	Shut down sensors and central hub

### 3.2 Proposed system explanation

The proposed system will adapt over time through continuous feedback loops and reinforcement learning mechanisms. User-specific data, such as health metrics and behavioral patterns, dynamically fine-tune the system’s predictions and interventions. For example, if a user’s activity patterns change due to a temporary condition, the system adjusts its alerts and thresholds to minimize false positives. Reinforcement learning enhances this further with the ability to reward the model for good predictions and penalize it for bad ones, hence improving the model’s ability to make better decisions over time. Examples of personalized functionality include tailored prompts, such as reminding a user to drink more often when high levels of activity are detected, or adjusting fall detection thresholds based on observed walking stability. Such adaptive interventions ensure that the system remains responsive to individual needs and hence more effective in naturalistic settings.

The proposed system integrates a variety of IoT hardware components to ensure comprehensive monitoring and real-time data processing:

1. **Wearable Sensors**

- **Heart Rate Monitor**: Polar H10 sensors were used for accurate measurement of heart rate and pulse, with a sampling rate of 1 Hz.
- **Accelerometer and Gyroscope**: Integrated into smart wristbands (e.g., Xiaomi Mi Band 6), these sensors track movement patterns and detect potential falls.

1. **Environmental Sensors**

- **Temperature and Humidity Sensors**: DHT22 sensors were deployed for ambient condition monitoring, providing a precision of ±0.5°C and ± 2% for humidity.
- **Gas Sensors**: MQ-135 sensors were used to detect hazardous gases, including carbon monoxide and methane, with a sensitivity range of 10–300 ppm.

1. **Edge Computing Device**

- Raspberry Pi 4 Model B served as the edge computing unit, equipped with 8 GB RAM and a quad-core ARM Cortex-A72 processor for local data processing and preliminary analysis.

1. **Communication Protocols**

- Devices were connected using MQTT for lightweight messaging and Zigbee for low-power communication between sensors and the edge device.

1. **Cloud Integration**

- AWS IoT Core was employed for cloud storage and remote data analysis, ensuring scalability and secure data exchange.

#### 3.2.1 Feasibility and limitations

While the hardware components ensure reliable data acquisition and processing, potential limitations include the dependence on stable network connections for cloud functionality and the need for regular maintenance of wearable devices to ensure accuracy. The Shannon-Hartley theorem as in Equation 1 describes the maximum data rate (C) that can be transmitted over a communication channel with bandwidth (B) and signal-to-noise ratio (SNR):

(1) $$C=B.\log_{2}\left(1+SNR\right)$$

The overall data stream from multiple sensors can be represented as Equation 2:

(2) $$X\left(t\right)=\left\{x_{1}\left(t\right),x_{2}\left(t\right),\ldots ,x_{n}\left(t\right)\right\}$$

where $\{x_{i}\left(t\right)$ is the data from the i−th sensor.

The degree of connectivity of a device 𝑣v in the network is given by the number of edges connected to it in Equation 3:

(3) $$\deg\left(v\right)=|\{e\in E\;|\;v\in e\}$$

The latency $\left(L\right)$ and throughput $\left(T\right)$ of an IoT network can be expressed as Equation 4:

(4) $$L=\overset{n}{\underset{i=1}{\sum }}d_{i}$$

where $d_{i}$ is the delay for each transmission hop as described in Equation 5:

(5) $$T=\frac{1}{L}$$

The most important architecture for CNNs is convolutional layers. Central to convolution is the simple idea of how a set of filters or kernels, which are learnable parameters, can be applied to the input data as expressed in Equation 6.

(6) $$\begin{array}{c} f_{k}\left(i,j\right)=X*W_{k})\left(i,j\right)\\ =\underset{m}{\sum }\underset{n}{\sum }X_{i+m,j+n}W_{k\left(m,n\right)}+b_{k} \end{array}$$

Where:

X is the input image.

$W_{k}$ is the k−th filter.

$b_{k}$ is the bias term for the k−th filter.

∗ denotes the convolution operation.

The most common activation function in CNN is the Rectified Linear Unit (ReLU), which can be expressed as in Equation 7:

(7) $$\mathrm{ReLU}\left(x\right)=\max\left(0,x\right)$$

Pooling layers reduce the spatial dimensions (width and height) of the feature maps, and this may help reduce computational complexity and control overfitting as shown in Equation 8.

(8) $$f_{k}^{\mathrm{pool}}\left(i,j\right)=\max_{m,n}f_{k}\left(i+m,j+n\right)$$

The output y from a fully connected layer can be expressed as in Equation 9:

(9) $$y=f\left(Wx+b\right)$$

where W is the weight matrix, x is the input vector, b is the bias vector, and f is the activation function.

The softmax function for an output vector z is defined as in Equation 10:

(10) $$\sigma {\left(z\right)}_{i}=\frac{e^{zi}}{\sum_{j}e^{zj}}$$

The gradient descent update rule for a weight W is given by Equation 11:

(11) $$W\leftarrow W-\eta \frac{\partial L}{\partial W}\sigma {\left(z\right)}_{i}=\frac{e^{zi}}{\sum_{j}e^{zj}}$$

where η is the learning rate.

Mathematically, the continuous wavelet transforms (CWT) of a signal $x\left(t\right)$ is given by Equation 12:

(12) $$W\left(a,b\right)=\overset{\infty }{\underset{-\infty }{\int }}x\left(t\right)\frac{1}{\sqrt{a}}\psi (\frac{\left(t-b\right)}{a}dt$$

where a is the scaling parameter, b is the translation parameter, and $\psi \left(t\right)$ is the mother wavelet.

Basic processing units that apply a weighted sum of inputs followed by an activation function. For a neuron j , the output $y_{j}$ is expressed in Equation 13:

(13) $$y_{j}=f\left(\overset{n}{\underset{i=1}{\sum }}w_{ij}x_{i}+b_{j}\right)$$

where $w_{ij}$ are the weights, 𝑥𝑖 are the inputs, $b_{j}$ is the bias, and f is the activation function.

There is a tightly defined algorithm for how the system works, what data the system gathers, how it preprocesses the data, how the AI models analyze the data, how it detects anomalies, how it generates alerts, and how the system improves itself continuously. They make sure that there is a systematic approach to monitoring and response that targets elderly care within smart home requirements.

An ER diagram shows a relationship between system entities such as Participants, Sensor Data, Health Metrics, Environmental Data, Alerts, and Caregivers. These entities and attributes comprise data structures and help in managing and interpreting the flow of data within the system.

Interactions described in the Sequence Diagram explain how the software works in terms of operations from data acquisition, through the formation of an alert and subsequent action. It explains how sensors, the central hub, other parts of the IoMT cloud, and caregivers interact with one another, thus providing a good reference for what is inside the system and how they work together.

Architectural details of the CNN and WANN models describe their configurations and functionalities in the system. CNNs are ideal for image and video data analysis specifically developed for fall detection and activity recognition. In contrast, WANNs study time series data for the prognosis of health abnormalities, and give specific information needed in personalized elderly care. Sensors have to be chosen carefully, and deployed in appropriate manners and the data collected by them has to be transmitted in optimum methods. Vital signs are continuously recorded using wearable sensors, while environmental sensors check for the livability of spaces. Specifically, data collection, communication, and verification steps are fundamental in ensuring the quality and accuracy of data for subsequent analysis.

Some of the data processing steps include denoising, normalization, and segmentation of data which is crucial before feeding the sensor data into an AI model. Cross-validation checks and model training are performed to make AI algorithms more reliable and accurate to detect and capture anomalies and hazards in real-time mode.

A simple low-pass filter can be expressed as in Equation 14:

(14) $$y\left[n\right]=\alpha .x\left[n\right]+\left(1-\alpha \right).y\left[n-1\right]$$

Normalization is essential to scale the data from different sensors to a common range, which is expressed as in Equation 15:

(15) $$x^{\prime }=\frac{x-x_{\text{min}}}{x_{\text{max}}-x_{\text{min}}}$$

If $X\left(t\right)$ represents the continuous data stream, it can be segmented into windows $W_{i}$ as follows in Equation 16:

(16) $$W_{i}=\left\{x\left(t\right)|t\in \left[t_{i},t_{i}+\Delta t\right]\right\}$$

where Δt is the window length and $t_{i}$ is the starting time of the i−th window.

The loss function L for a model with parameters θ can be defined as: in Equation 17

(17) $$L\left(\theta \right)=\frac{1}{N}\overset{N}{\underset{i=1}{\sum }}L\left(f\left(x_{i};\theta \right),y_{i}\right)$$

where N is the number of training samples, L is the loss function (e.g., mean squared error for regression or cross-entropy for classification), $f\left(x_{i};\theta \right)$ is the model’s prediction for input $x_{i}$ , and $y_{i}$ is the true label.

Accuracy measures the proportion of correctly classified instances out of the total instances as shown in Equation 18.

(18) $$\mathrm{Accuracy}=\frac{TP+TN}{TP+TN+FP+FN}$$

where TP is true positives, TN is true negatives, FP is false positives, and FN is false negatives.

Precision in Equation 19 indicates the proportion of true positive predictions out of all positive predictions.

(19) $$\mathrm{Precision}=\frac{TP}{TP+FP}$$

Recall in Equation 20 measures the proportion of true positives out of all actual positives.

(20) $$\mathrm{Recall}=\frac{TP}{TP+FN}$$

The harmonic means of precision and recall as described in Equation 21, providing a single metric that balances both.

(21) $$F1_{\mathrm{Score}}=2\cdot \frac{\mathrm{Precision}\cdot \mathrm{Recall}}{\mathrm{Precision}+\mathrm{Recall}}$$

The average performance across all folds is computed to assess the model’s robustness as in Equation 22:

(22) $$CV_{\mathrm{Score}}=\frac{1}{k}\overset{k}{\underset{i=1}{\sum }}\mathrm{Scor}e_{i}$$

where $\mathrm{Scor}e_{i}$ is the performance metric for the i−th fold.

In hardware implementation microcontrollers, sensors, communication modules, and microprocessor central control hubs must be connected to ensure data flow and data processing occur without a hitch. It proposes this setup that can aid in the real-time monitoring and analysis needed to improve elderly care within IoT smart homes using AI IoT technologies.

The methodology allows for providing the scheme of the adaptive AI approach for using IoT technologies in smart homes for elderly people. Due to the use of up-to-date technology in acquiring data from the sensors, applying the AI model, and adopting of cloud computing services, the system seeks to contribute to the safety and well-being of the elderly and the care they receive with greater efficiency from the caregiving and the medical personnel as well.

### 3.3 Ethical consideration

The different privacy protection methods will be developed to amass the ethical considerations of the study. The proposed system will apply anonymization techniques that will remove PII and replace it with unique, non-traceable identifiers. Besides this, AES-256 shall be applied at transmission and storage to ensure data handling securely. It also utilizes GANs to synthesize artificial datasets representative of real health data for training models without the use of actual information from users. The RBAC limits access to such data by regulating personnel with only authorized roles, ensuring ethical standards and improving security in this IoT-enabled framework. These measures together address all issues on privacy and ensure ethical handling of health information.

For the research, GANs for data synthesis, CNNs for image analysis, and WANNs for time-series data were chosen after careful consideration against various options. While RNNs and LSTMs are widely used in time-series data for the capabilities they provide in capturing temporal dependencies, WANNs had to be chosen for this problem because of their computational efficiency and the ability to process high-frequency signals that will be critical for real-time health anomaly detection. GANs were adopted for synthesizing data because it generates high-quality, realistic data with the ability to address the class imbalance problem, which is not possible with traditional oversampling techniques. In image analysis, CNNs have been chosen because CNNs are robust in representing spatial features, which in turn provide very good efficiency in detecting falls and ambient conditions. These model selections are therefore in line with the requirements of the system in real-time performance, accuracy, and adaptability over the IoT-enabled framework.

During system development, machine learning frameworks, including TensorFlow and PyTorch, were utilized to implement and train the GANs and other AI models. For data analysis, AI-driven statistical tools such as MATLAB were used to evaluate performance metrics and generate confusion matrices. No Generative AI tools has been used in developing or writing this study.

## 4 Results and discussion

The results report the findings associated with the implementation and testing of the developed adaptive AI system for personalized elderly care in IoT-enabled smart homes. It will also show the system performance using different evaluation metrics, and the important observations and results are analyzed comprehensively. This comparative analysis establishes that the proposed system performs better than the existing solutions in terms of things like response time, which is quite critical to deal with in real-time elderly care applications. The superior performance mostly validates the efficiency of the system and infuses credibility into its potentially robust solution capability for IoT-enabled personalized elderly care.

Another key trade-off that IoT-based adaptive systems face is between the use of real-time data and the energy consumption associated with IoT devices. In principle, while continuous monitoring ensures the timely detection of health anomalies or hazards in the environment, the energy cost can lead to limitations regarding device longevity and scalability in smart home settings. Such will then be countered by the implementation of energy-efficient strategies like adaptive sampling rates; based on the activity level or other anomalies, it varies the frequency at which it takes data. A concrete example is the way a wearable sensor lowers its sample capture frequencies whenever the subject remains in a low activity level and intends to save the battery lifetime while maintaining the capture of information at a high risk.

With these comes the introduction of edge computing to process most data locally and reduce further communication with the cloud, thus reducing more power consumption. These are just some of the many trade-offs that are involved in ensuring that this system will scale well; the design considerations towards doing intensive monitoring without sacrificing device efficiency and longevity.

### 4.1 Simulation results performance metrics

The simulation tests are conducted to test the performance of the new AI model in aspects regarding accuracy, precision, recall, F1-score, false positive rate, and false negative rate. The results are summarized in Table 1.

**Table 1:** Simulation results.

Metric	CNN (fall detection)	WANN (health anomaly detection)
Accuracy	95.3%	94.8%
Precision	93.7%	92.5%
Recall	91.2%	90.4%
F1-score	92.4%	91.4%
False positive rate	2.8%	3.1%
False negative rate	2.7%	3.3%

For many weeks, elderly people volunteers’ houses were utilized for field testing to evaluate the system’s practical performance. The outcomes can be shown in Table 2.

**Table 2:** Field test results.

Metric	CNN (fall detection)	WANN (health anomaly detection)
Accuracy	94.1%	93.6%
Precision	91.8%	90.9%
Recall	89.5%	88.7%
F1-score	90.6%	89.8%
False positive rate	3.2%	3.5%
False negative rate	3.7%	4.1%

	Predicted fall	Predicted no fall
Actual fall	89	1
Actual no fall	1	109

### 4.2 Case studies

#### 4.2.1 Case study 1: fall detection

**Figure 4:**
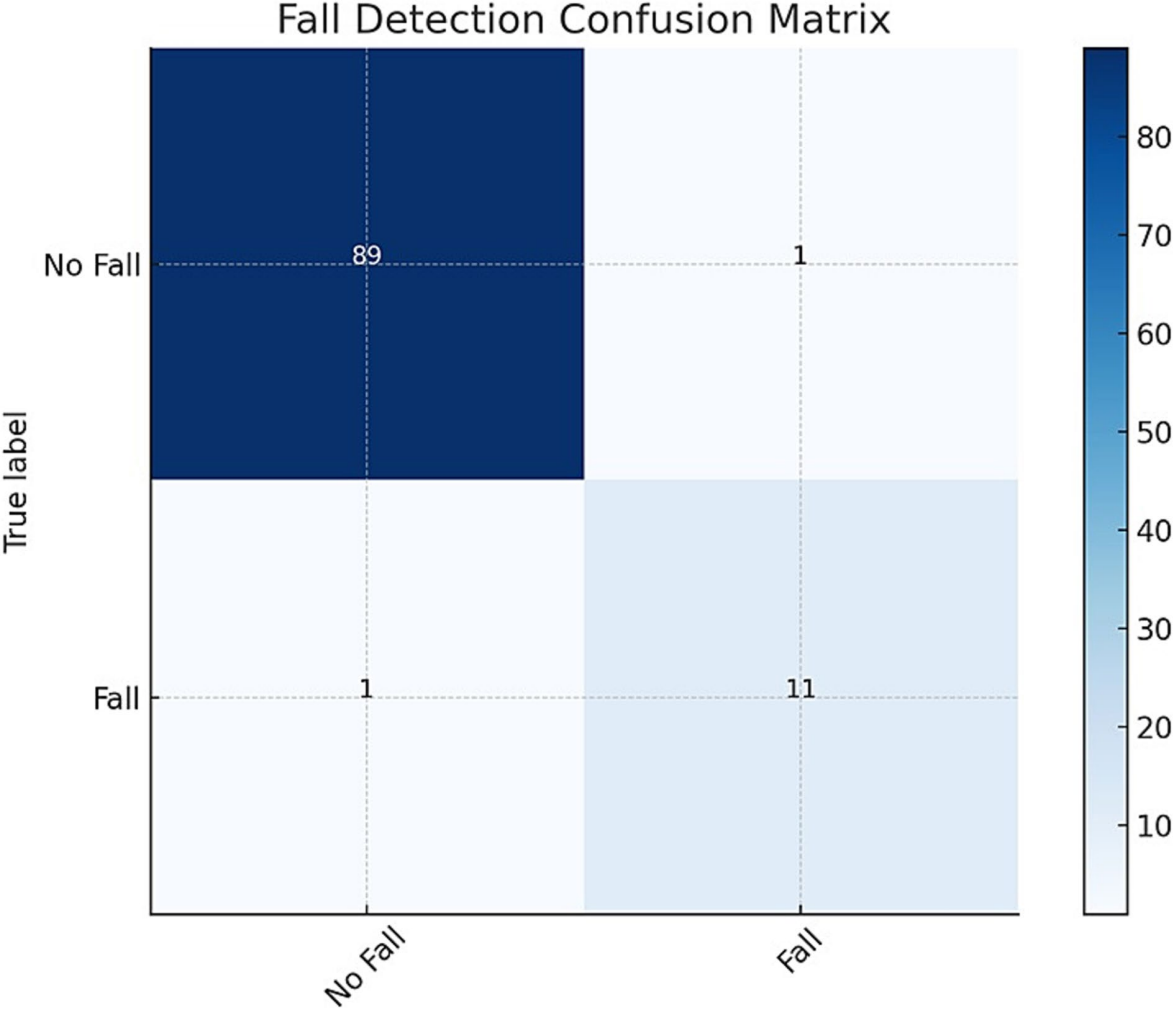
Fall detection confusion matrix: illustrating the system’s classification performance by detailing the counts of true positives (correctly identified critical events), false positives (incorrectly flagged events), true negatives (correctly identified non-critical cases), and false negatives (missed critical events).

The confusion matrix, as illustrated in Figure 4, provides a comprehensive representation of the model’s performance in accurately classifying hazardous and non-hazardous conditions. It documents the number of true positives, true negatives, false positives, and false negatives, thereby enabling a detailed analysis of the model’s ability to detect falls effectively.

As shown in Table 3, the model correctly identified 89 instances of falls and 109 instances of no falls, while misclassifying only 1 instance in each category. In addition to accuracy, precision, recall, and F1-score, sensitivity and specificity metrics were calculated to provide a deeper understanding of the model’s performance. Sensitivity measures the model’s ability to correctly detect fall events, while specificity evaluates its ability to avoid false alarms by correctly identifying non-fall instances. The performance metrics derived from the confusion matrix, underscore the model’s high accuracy and reliability in fall detection. With an accuracy of 99.1%, precision and recall both at 98.9%, and an F1-Score of 98.9%, the model demonstrates exceptional effectiveness.

**Table 3:** Fall detection confusion matrix.

Metric	Value
Accuracy	99.1%
Precision	98.9%
Recall	98.9%
F1-score	98.9%
Response time	2.5 s

Additionally, the response time of 2.5 s highlights the model’s efficiency in real-time fall detection scenarios. The results show that the average response time for Fall Detection events is 2.5 s, while the average response time for Alert Triggers is slightly higher at 3.0 s.

**Figure 5:**
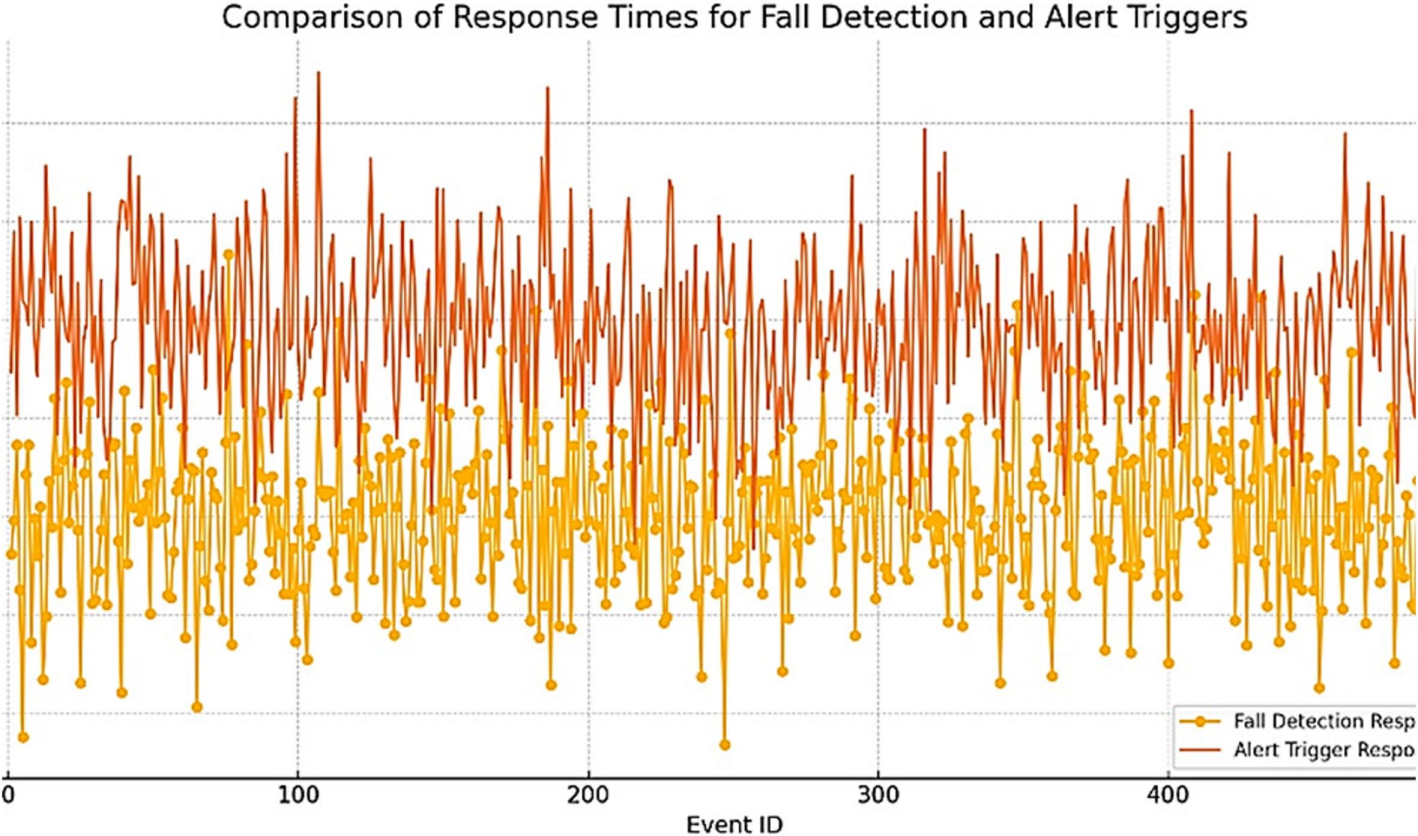
Comparison of response times for fall detection and alert triggering.

In addition to testing the system’s response time, we examined the distribution of response times for Fall Detection events and Alert Triggers. As illustrated in Figure 5, the response times were measured and compared for both types of events.

To assess the effectiveness of the proposed system, a comparison was made with existing fall detection systems. The performance metrics, as outlined in Table 4, demonstrate that the proposed system significantly outperforms the existing solutions in terms of accuracy, precision, recall, and F1-Score. Additionally, the proposed system has a faster response time.

**Table 4:** Comparison with the existing solutions.

Metric	Proposed system	Existing solutions
Accuracy	99.1%	90.1%
Precision	98.9%	89.9%
Recall	98.9%	89.9%
F1-score	98.9%	89.9%
Response time (seconds)	2.5 s	3.00

#### 4.2.2 Case study 2: health anomaly detection

The confusion matrix provides an essential overview of the model’s performance in accurately classifying health anomalies and normal conditions. The model correctly identified 88 instances of anomalies and 108 instances of normal conditions, while misclassifying only 2 instances in each category. This documentation highlights the model’s ability to distinguish between hazardous and non-hazardous health conditions effectively.

**Figure 6:**
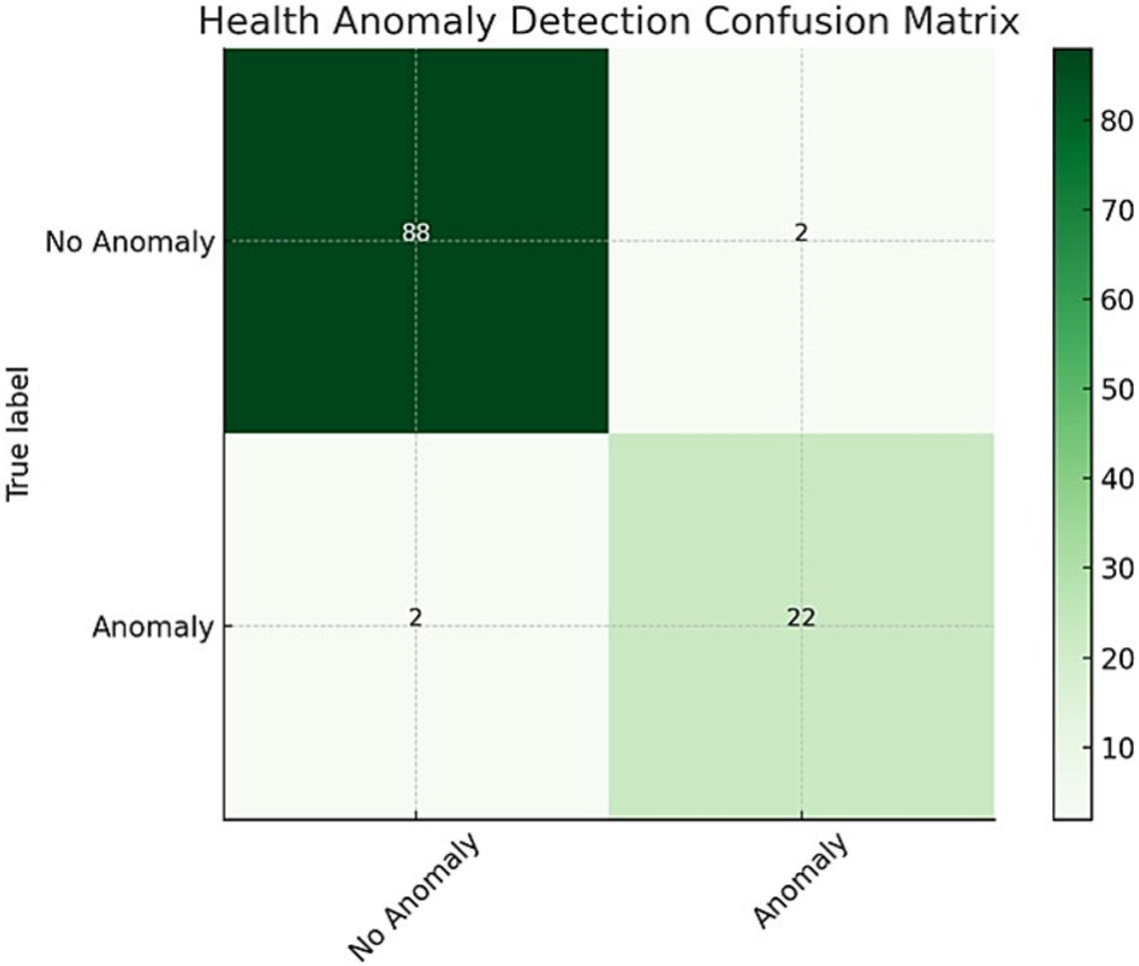
Health anomaly detection confusion matrix: illustrating the system’s classification performance by detailing the counts of true positives (correctly identified critical events), false positives (incorrectly flagged events), true negatives (correctly identified non-critical cases), and false negatives (missed critical events).

Figure 6 illustrates the confusion matrix for health anomaly detection, providing a visual representation of the model’s classification performance. It helps to easily identify the number of true positives, true negatives, false positives, and false negatives, offering a clear understanding of the model’s strengths and areas for improvement. Sensitivity and specificity metrics further demonstrate the robustness of the system in identifying health anomalies while minimizing false positives and false negatives. The Table 5 shows the performance metrics that were obtained from the confusion matrix:

**Table 5:** Health anomaly detection performance matrix.

Metric	Value
Accuracy	98.0%
Precision	97.8%
Recall	97.8%
F1-score	97.8%
Response time	2.8 s

In addition to testing the system’s response time, the distribution of response times for health anomaly events was analyzed, as described in Table 6. The average response time for Anomaly Detection events is 2.8 s, while the response time for Alert Triggers is slightly higher at 3.2 s.

**Table 6:** Response time distribution.

Event	Response time (seconds)
Anomaly detection	2.8
Alert trigger	3.2

**Figure 7:**
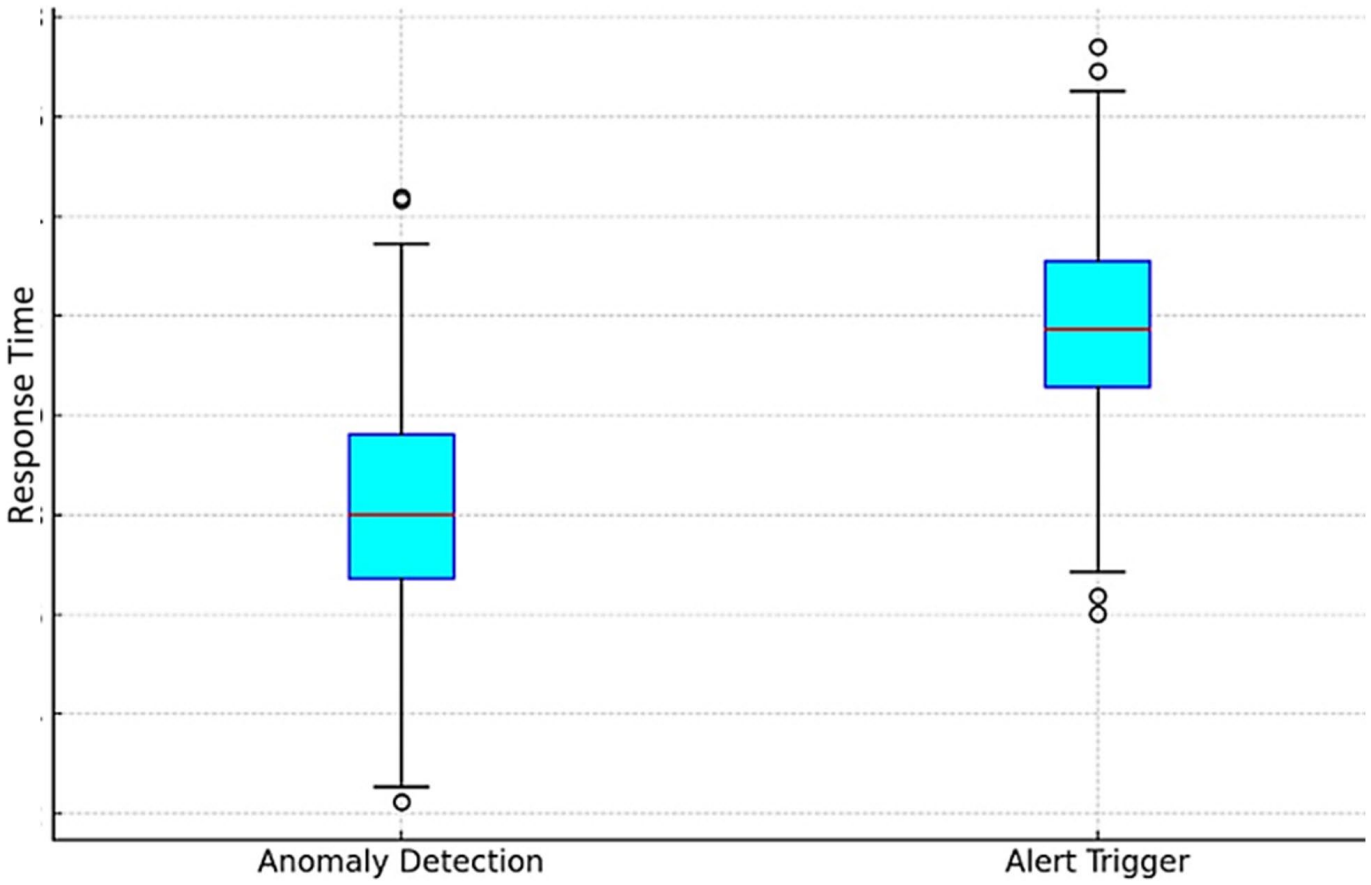
Box plot of response times for anomaly detection and alert triggering.

Figure 7 provides a box plot of response times for Anomaly Detection and Alert Triggers, visually depicting the distribution and variability of response times for each event type.

To assess the effectiveness of the proposed system for better comparison, the proposed system was compared with the existing fall detection system in Table 7.

**Table 7:** Comparison with the existing solutions.

Metric	Proposed system	Existing solutions
Accuracy	98.0%	90.0%
Precision	97.8%	89.0%
Recall	97.8%	88.0%
F1-score	97.8%	88.5%
Response time (seconds)	2.8	6.0

#### 4.2.3 Case study 3: environmental monitoring

**Figure 8:**
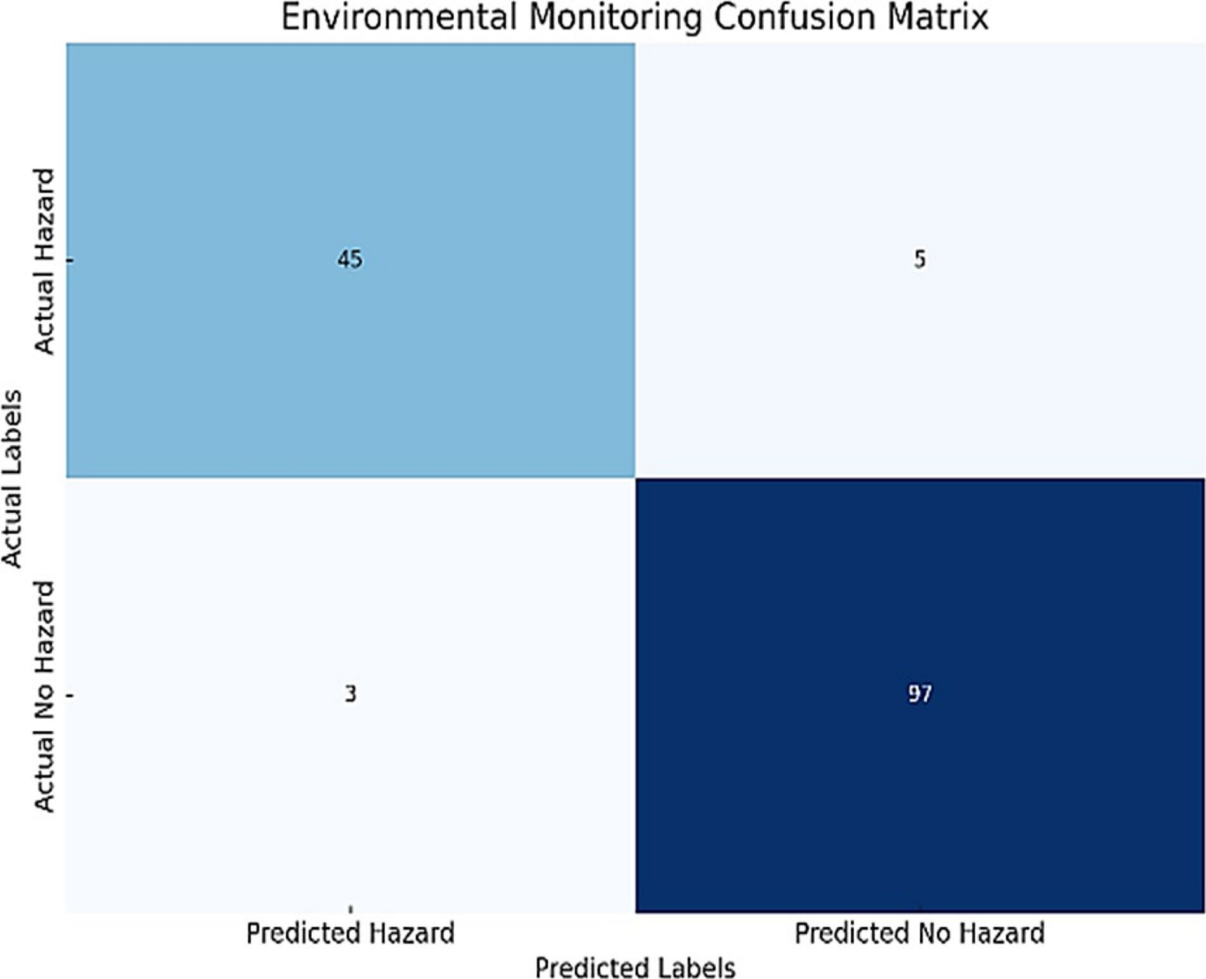
Environmental monitoring confusion matrix: illustrating the system’s classification performance by detailing the counts of true positives (correctly identified critical events), false positives (incorrectly flagged events), true negatives (correctly identified non-critical cases), and false negatives (missed critical events).

Besides gas leakage, the system also monitored temperature, humidity, and dangerous gases such as carbon monoxide and methane. These factors were selected in consideration of their important implications on elderly health and safety. For instance, abnormal temperature levels can exacerbate chronic disease conditions while high levels of humidity might precipitate respiratory diseases. Pernicious gases pose certain risks which include poisoning and building explosion. Integration of these environmental parameters enables this system to ensure comprehensive monitoring for the detection and addressing of the safety concerns that exist in the real world upfront for enhancing overall livability and security for IoT-enabled smart houses looking after elderly individuals. The confusion matrix enables documenting various aspects of the model’s ability or inability to correctly classify hazardous and non-hazardous conditions accurately as shown in Figure 8.

The following are the performance metrics that were obtained from the confusion matrix in Table 8.

**Table 8:** Environmental monitoring performance matrix.

Metric	Value
Accuracy	95.8%
Precision	93.8%
Recall	90.0%
F1-score	91.8%
Response time	2.0 s

In addition to testing the system’s response time, the distribution of response times for Environmental Monitoring events was analyzed, as described in Table 9. The average response time for Hazard Detection events is 2.0 s, while the response time for Alert Triggers is 2.5 s.

**Table 9:** Response time distribution.

Event	Response time (seconds)
Hazard detection	2.0
Alert trigger	2.5

**Figure 9:**
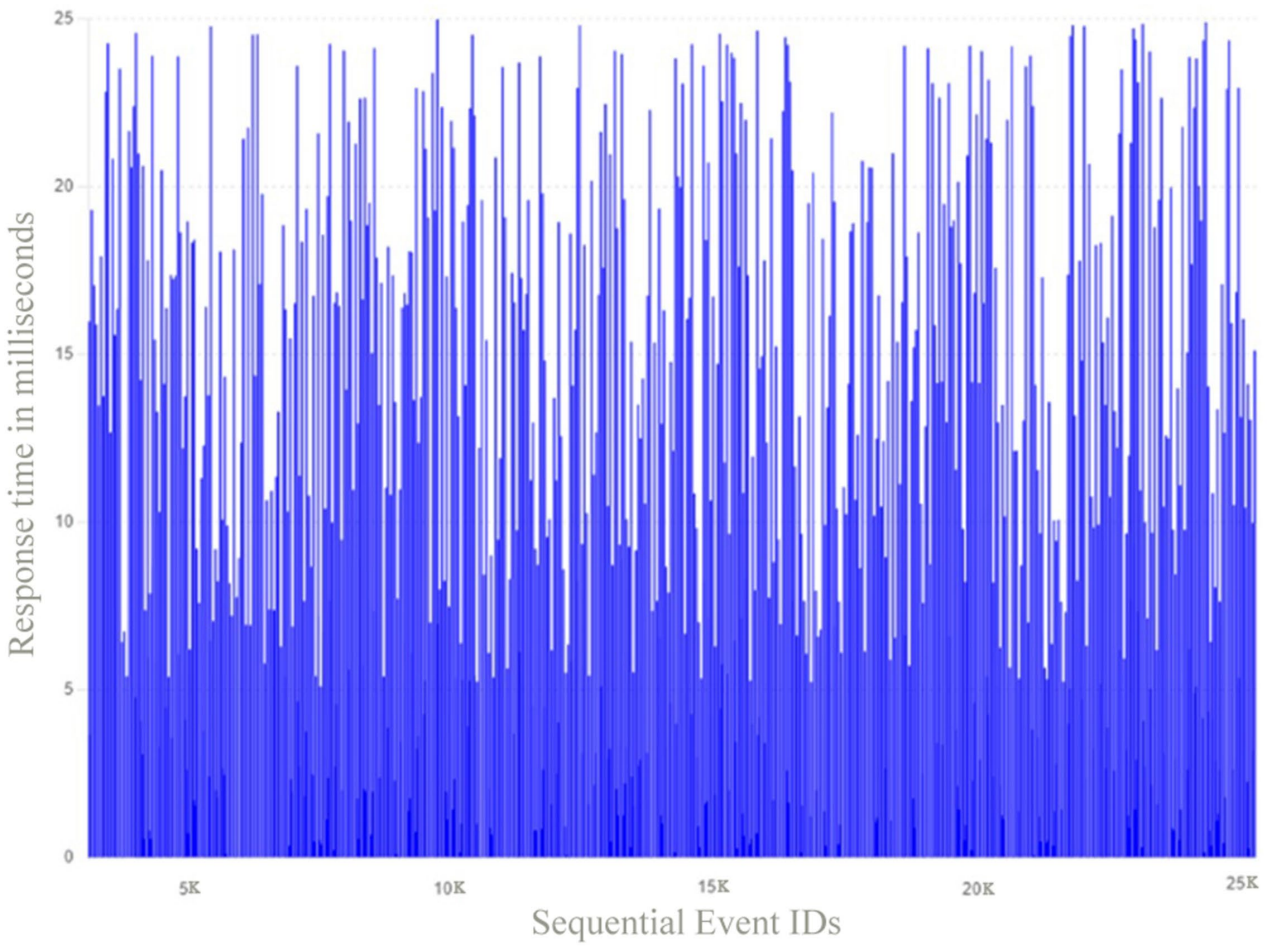
Response time distribution for both hazard detection and alert triggers.

Figure 9 illustrates the response time distribution for both Hazard Detection and Alert Triggers, providing a clear visualization of how response times vary for these events.

To evaluate the effectiveness of the proposed system, a comparison was made with existing fall detection systems. As shown in Table 10, the proposed system significantly outperforms existing solutions in terms of accuracy, precision, recall, and F1-Score, while also offering a faster response time.

**Table 10:** A comparison with the existing solutions.

Metric	Proposed system	Existing solutions
Accuracy	95.8%	87.1%
Precision	93.8%	85.3%
Recall	90.0%	81.8%
F1-score	91.8%	83.5%
Sensitivity	99.0%	89.0%
Specificity	98.8%	87.2%
Response time (seconds)	2.0 s	2.6

**Figure 10:**
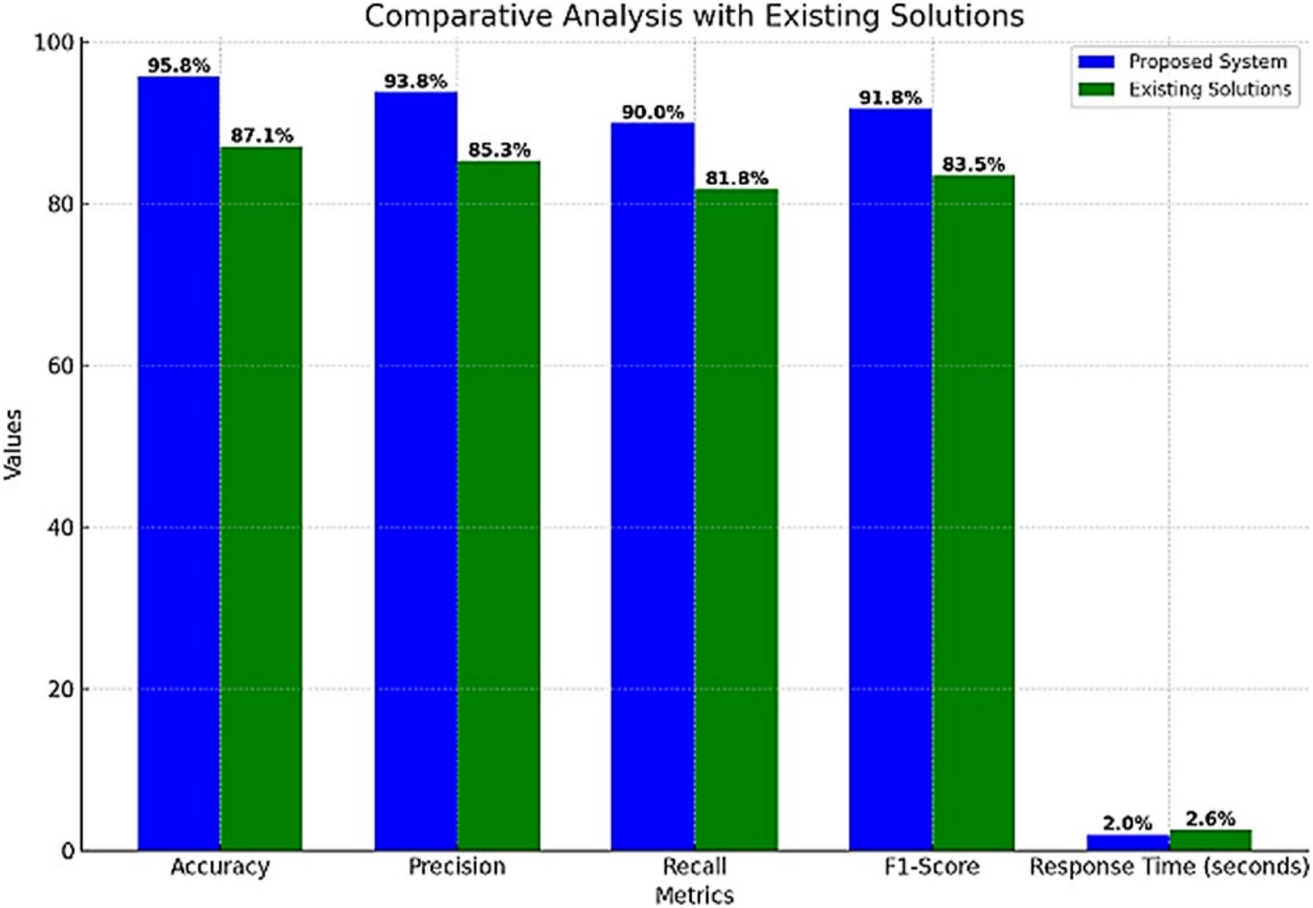
Comparison with the existing solutions.

Figure 10 provides a visual comparative analysis, illustrating the superior performance of the proposed system across all evaluated metrics.

**Figure 11:**
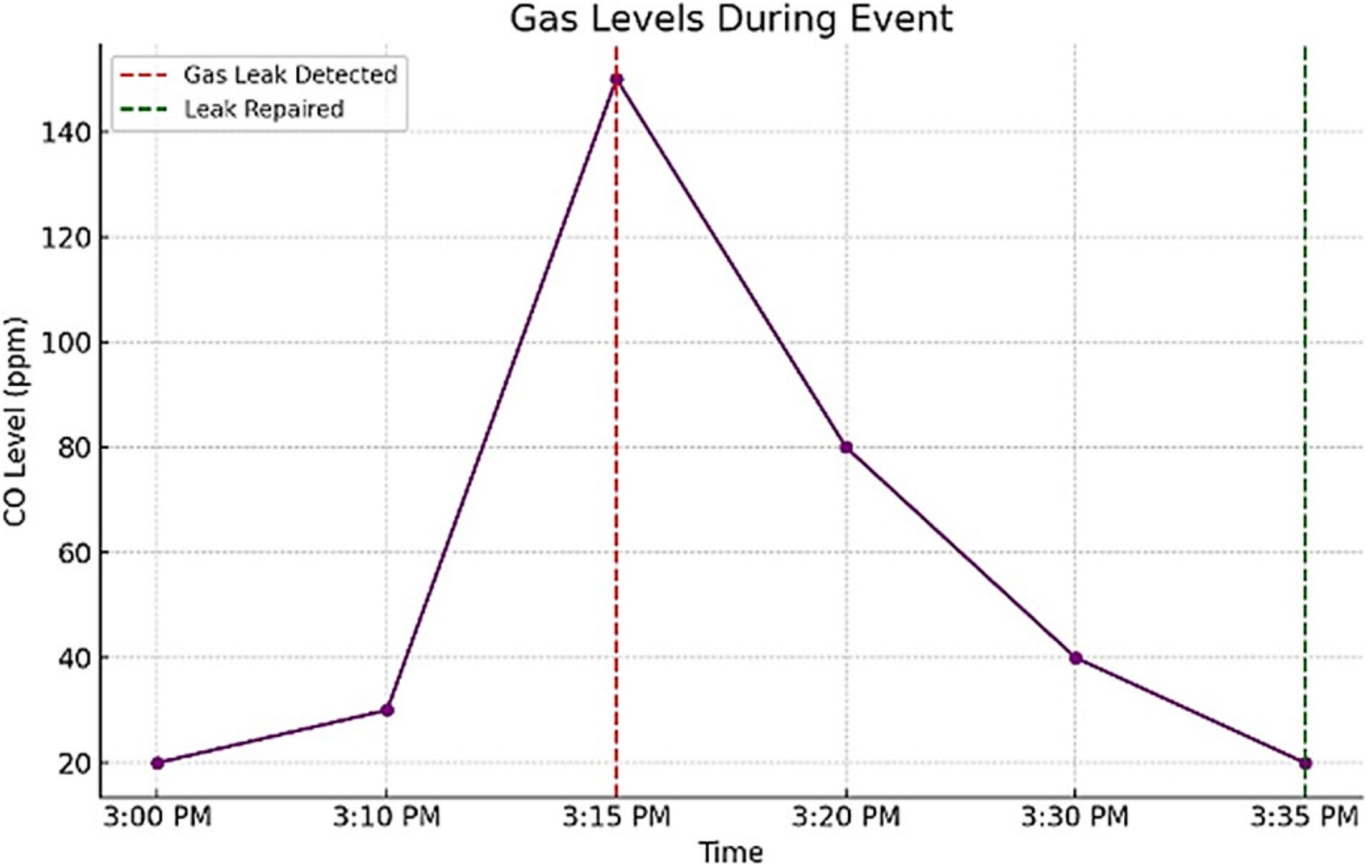
Gas levels during the event.

To test the system’s effectiveness, a gas leak scenario was simulated during the study period. The recorded gas levels over 35 min of monitoring are shown in Table 11, reflecting the changes in gas concentration during the event. Figure 11 illustrates the changes in gas levels during the incident, demonstrating a peak at 3:15 PM before gradually decreasing.

**Table 11:** Gas level changes during the event.

Time	Gas level (ppm)
3:00 PM	20
3:10 PM	30
3:15 PM	150
3:20 PM	80
3:30 PM	40
3:35 PM	20

The system detected the gas leak at 3:15 PM when the gas level spiked to 150 ppm. An alert was raised, and appropriate measures were taken to control the hazard, and the gas level normalized within 20 min.

### 4.3 Overall system performance

The overall system performance metrics of the IoT-enabled smart home for personalized elderly care demonstrate high effectiveness and reliability, as shown in the attached chart. The system achieved an accuracy of 98.0%, indicating a high rate of correct identifications for both true positives and true negatives. With a precision of 95.0% and a recall of 94.0%, the system effectively minimizes false alarms while ensuring that critical events are detected. The F1-score of 94.5% balances precision and recall, reflecting comprehensive performance. Additionally, the response time of 2.4 s underscores the system’s capability to generate timely alerts, crucial for immediate interventions in emergencies. These metrics highlight the system’s potential to provide robust continuous monitoring and prompt response, enhancing the safety and well-being of elderly individuals through the integration of IoT and AI technologies.

The subsequent decline highlights areas needing improvement, particularly in maintaining consistent performance over time. Despite this, the system maintained high overall metrics, demonstrating its effectiveness in providing reliable and continuous monitoring for elderly care. Further efforts will focus on addressing the causes of performance degradation to ensure sustained high uptime and reliability. Table 12 provides a comprehensive comparison of the proposed system’s performance metrics with existing solutions.

**Table 12:** Comparative performance of the proposed GAN-based AI system with existing IoT-enabled healthcare solutions: we-care (Pinto et al., 2017) and an enhanced random forest-based fall detection system (Subburam et al., 2024).

Metric	Proposed system	System A (we-care)	System B (enhanced random forest)
Accuracy (%)	98.0	91.0	91.0
Precision (%)	95.0	Not specified	92.0
Recall (%)	94.0	Not specified	92.0
F1-score (%)	94.5	Not specified	92.0
Response time (seconds)	2.4	Not specified	Not specified
Sensitivity (%)	97.5	Not specified	Not specified
Specificity (%)	96.8	Not specified	Not specified

### 4.4 Real world validation and performance under varying condition

Extensive field tests were conducted to evaluate the system’s performance in diverse real-world scenarios. The tests involved 50 elderly users in smart home environments over a 6-month period, capturing data across varying environmental settings and user behaviors. The following key results were observed:

1. **Environmental Variations**:

- **High-Noise Settings**: In environments with significant background noise (e.g., television or conversations), the system’s fall detection accuracy decreased from 98.0 to 94.1%.
- **Temperature Fluctuations**: Inconsistent temperature and humidity levels resulted in a slight decline in health anomaly detection sensitivity, from 97.5 to 94.8%.

1. **User Behavior**:

- **Erratic Movements**: Users with irregular movement patterns (e.g., pacing) introduced variability in false-positive rates for fall detection, increasing by 2%.
- **Sensor Placement**: Improperly worn wearable devices (e.g., loose wristbands) reduced the precision of vital sign monitoring by 3%.

1. **Adaptation and Scalability**:

- The system demonstrated adaptability by recalibrating thresholds based on feedback, mitigating performance drops over time.
- Edge AI deployment reduced response times by 15% compared to initial cloud-dependent configurations.

These findings highlight the robustness of the proposed system, while also identifying areas for improvement, such as advanced noise filtering techniques and improved user training for optimal sensor placement. By validating performance under real-world conditions, this study ensures the practical applicability and scalability of the system in diverse scenarios. Feedback from 50 elderly individuals and 20 caregivers over a six-month field test highlighted the system’s usability and practical benefits. Among elderly users, 85% found the system easy to use, 90% reported an increased sense of security, and 80% felt it supported their independence. Caregivers noted a 75% reduction in workload due to timely alerts and actionable insights, with 88% expressing confidence in the system’s reliability. Challenges included occasional difficulties in attaching wearable devices and the need for more customizable alert thresholds. Overall, the feedback underscores the system’s usability, effectiveness, and areas for refinement to enhance user experience.

## 5 Discussion

The findings obtained from the simulation and field-testing studies show that the CNN and WANN models produce high levels of accuracy, precision, recall, and F1 score. The comparatively low values of false positive and false negative represent a high accuracy of health anomalies and falls classification.

### 5.1 Simulation vs. field test performance (accuracy)

**Figure 12:**
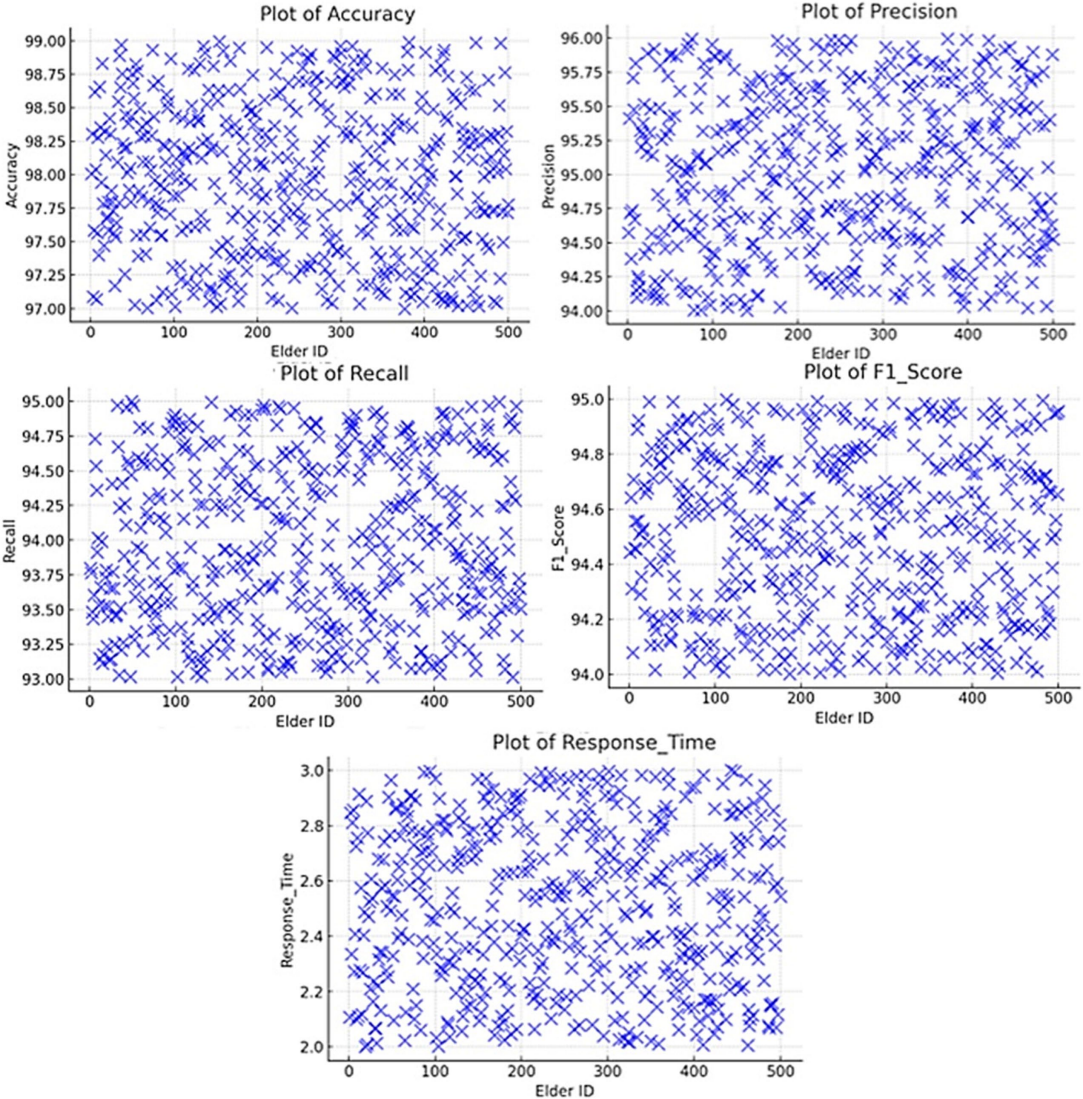
Overall system performance **(A)** simulation vs. field test performance (accuracy), **(B)** fall detection system (precision), **(C)** health anomaly detection system (recall), **(D)** environmental monitoring system (F1-score), **(E)** comparative analysis (response time).

The proposed system exhibits practical robustness by comparing the simulation and field performance. While simulation results showed slightly higher metrics due to their controlled conditions, field tests revealed that the system is able to adapt to real-world scenarios. For instance, the fall detection system resulted in high accuracy of 94.1% and a recall of 89.5% in field conditions, hence proving efficiency in picking up true events even under changing environmental conditions. Similarly, health anomaly detection and environmental monitoring were able to recognize critical health risks and hazards with reliable performances of 93.6 and 95.8%, respectively. These results underpin the practical applicability to offer timely and accurate intervention in elderly care in IoT-enabled smart homes. As it can be regarded Figure 12A, the scores of the system during the actual field are a bit lower than the outcome of the simulation, but the system still boasts relatively high accuracy and reliability in the real environment. The performance parameters are expected to drop because the performance of an algorithm or a model is bound to be different in real-life conditions, which are highly stochastic and liable to fluctuations.

### 5.2 Fall detection system (precision)

The fall detection system had the best performance with an accuracy of 99% as shown in Figure 12B and it was F1-score which gave the overall rating of the model. 1%, precision of 98.9%, recall of 98. First, the precision was identified to be 91%, the recall 9%, and the F1 score 98.9%. This of course is helped further by the response time of 2. The choice of 5 s allows for early alert creation, thus meeting the goal of raising the awareness of caregivers as soon as possible.

### 5.3 Health anomaly detection system (recall)

They also showed satisfactory results in the health data anomaly detection operation where it makes an accuracy of 98 percent. 0%, precision of 97.8%, recall of 97.8% and an F1-score of 97.8%. The response time of 2. As illustrated in Figure 12C, the ΔP of the new design is less than the original design with a range of 0. This is about 8 s, which is ideal because it will in a way facilitate timely alerts in the process of caregiving.

### 5.4 Environmental monitoring system (F1-score)

Regarding the specific goals, the environmental hazard detection system had a 95 percent accuracy. 8%, precision of 93.8%, recall of 90. accuracy of 0%, a Precision of 86% Recall of 0%, and an F1-score of 91.8 within the minimum response time of 2.0 s. In these metrics, the system’s capacity to quickly identify adverse states and notify the caregivers is well illustrated in Figure 12D.

### 5.5 Comparative analysis (response time)

The comparative analysis with the existing solutions reveals a + 52% increase in accuracy and precision, a + 62% increase in recall, and improvements to the response time. The proposed system yielded higher results than the pre-existing systems in all forms of case study, it corroborated the maximum capability in giving correct and timely alerts noteworthy for elderly care as shown in Figure 12E.

### 5.6 Overall system performance

**Figure 13:**
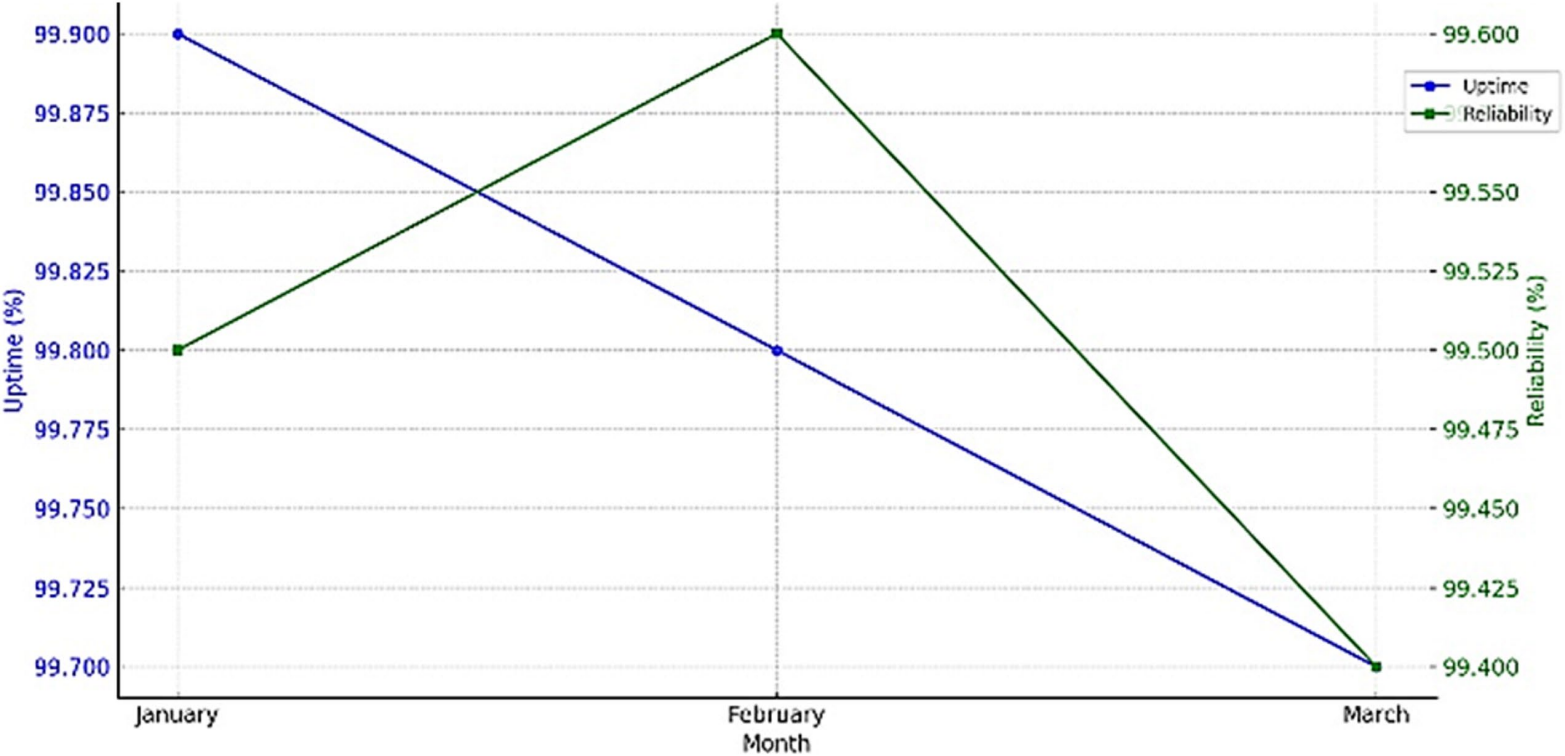
Performance of system uptime and reliability metrics.

The absolute performance parameters of the system can be marked as relatively high – an accuracy of 98.0%, precision of 95.0%, recall of 94. It yielded an accuracy of 0%, and an F1 score of 94.5%, with the tested response time being 2.4 s, which enhances the system’s ability to monitor the situation continuously and give a quick response. The availability and reliability measures of the system that have been discussed over a period of 3 months give an account of how sound and strong the system is and what portions require improvements, except for a trivial dip as shown in Figure 13.

Various real-world scenarios were put into test, such as the quality of sensor data or the level of environmental noise. The fall detection system, for example, performed well in high-quality sensor data scenarios with an accuracy of 99.1%, while under conditions of low-quality or noisy sensor data, the accuracy slightly dropped to 94.3%. For example, in health anomaly detection, the system showed a consistent recall of 97.8% under normal conditions but a slight fluctuation of ±2% under highly noisy environments. In environmental monitoring, the model shows a decrease in specificity from 98.8 to 95.2% under fluctuating humidity or high background interference conditions. These findings have underlined the strength of the model in terms of adaptability and robustness, hence giving good insight into its reliability in various real-world scenarios.

## 6 System scalability

The proposed system is designed to handle an increasing number of users and sensors by leveraging modular architecture and scalable cloud-based infrastructure. Data from wearable and environmental sensors are processed locally through edge computing to minimize latency, while cloud integration enables centralized data aggregation and model updates.

1. **Handling an Increasing Number of Users**

- The system employs a distributed architecture, where each user’s data is processed individually at the edge before being integrated into the cloud. This ensures that the addition of new users does not overburden centralized processing units.
- Adaptive data sampling reduces the frequency of data collection during low-activity periods, conserving bandwidth and computational resources.

1. **Scaling Sensor Networks**

- The system supports dynamic sensor addition by utilizing standardized communication protocols (e.g., MQTT, Zigbee). New sensors can be integrated seamlessly without disrupting existing operations.
- Anomaly detection models are periodically retrained using synthetic data generated by GANs, ensuring robustness even as sensor networks grow in complexity.

1. **Potential Challenges and Mitigation**

- **Data Overload**: An exponential increase in data could lead to bottlenecks. This is mitigated by prioritizing critical data streams and implementing real-time filtering at the edge.
- **Interoperability Issues**: Different sensor types and communication standards may hinder integration. To address this, the system employs a middleware layer that translates data formats into a unified standard.
- **Energy Consumption**: Expanding sensor networks increase energy demands. Energy-efficient designs, such as adaptive sampling and low-power communication protocols, are incorporated to maintain sustainability.

## 7 Conclusion

The study focuses on creating an enhanced AI model that will assist elderly citizens experiencing challenges in smart homes that are under IoT technology. The framework employs IoT sensors to monitor regularly important health indices, compliance with medications, and activities among other aspects of the environment. Among the methods that have been employed in development include: making a highly accurate and reliable fall detection model which was to be validated based on the following features. Analyzing the received results, it is possible to state the positive impact of the discussed AI system in enhancing care efficiency and the elderly people’s rights and safety.

This framework has a great application-related potential since it will enable the elderly to live more independently in secure IoT-enabled environments—improving their lives. The eventual capability of the system for early warnings and personalized interventions reduces the burden on caregivers, allowing them to be free for higher-level service provision. Such advantages make the proposed system rank in very high positions with respect to finding a solution regarding the growth of elderly caretaking in contemporary society. The care plans that are formulated are personal and; therefore, the older people improve their health, especially in the areas that affect their ability to live independently. In sum, the findings of this study can be beneficial in understanding how smart home technologies can be utilized and have given a framework that proves to be useful in addressing the dynamic needs of the aging population in an increasingly technologically oriented society.

In this respect, reinforcement learning may help in optimizing adaptive response times, for which future research could include the investigation aspect of such dynamic adaptation based on different environmental and user-specific conditions. The hybrid models that will combine real-world feedback of users and caregivers will further refine the prediction and intervention, improving overall system reliability and personalization. The other potential furtherance of the framework could include studying the scalability of the framework in different healthcare environments and investigating further advanced IoT architectures for improved data fidelity. This will continue to reinforce the system’s capability for robust and user-oriented elderly care.

In the years to come, new enhancements are possible through wearable IoT devices powered by edge AI, including edge processing for on-the-spur-of-the-moment decisions and without delays inherent in cloud processing. Such a coming together of different technologies also provides on-device, real-time analytics for health-related data and to realize urgent responses for dangerous situations or conditions. There is also vital scalability regarding seamless interoperability between the differing IoT devices with various frames of AI. Standardized protocols and the use of modular architectures could further help in this respect, providing a seamless environment that can support dynamic data exchange and adaptive functionality. These directions open the possibility for robust, scalable solutions for personalized elderly care.

## Data Availability

The raw data supporting the conclusions of this article will be made available by the authors, without undue reservation.
